# Key clinical benefits of neuroimaging at 7 T

**DOI:** 10.1016/j.neuroimage.2016.11.031

**Published:** 2016-11-13

**Authors:** Siegfried Trattnig, Elisabeth Springer, Wolfgang Bogner, Gilbert Hangel, Bernhard Strasser, Barbara Dymerska, Pedro Lima Cardoso, Simon Daniel Robinson

**Affiliations:** a High Field MR Center, Department of Biomedical Imaging and Image-guided Therapy, Medical University of Vienna, Lazarettgasse 14, A-1090, Vienna, Austria; b Christian Doppler Laboratory for Clinical Molecular MRI, Vienna, Austria

**Keywords:** clinical, 7 T, neuroimaging, high resolution, MRSI, fMRI

## Abstract

The growing interest in ultra-high field MRI, with more than 35.000 MR examinations already performed at 7 T, is related to improved clinical results with regard to morphological as well as functional and metabolic capabilities. Since the signal-to-noise ratio increases with the field strength of the MR scanner, the most evident application at 7 T is to gain higher spatial resolution in the brain compared to 3 T. Of specific clinical interest for neuro applications is the cerebral cortex at 7 T, for the detection of changes in cortical structure, like the visualization of cortical microinfarcts and cortical plaques in Multiple Sclerosis. In imaging of the hippocampus, even subfields of the internal hippocampal anatomy and pathology may be visualized with excellent spatial resolution. Using Susceptibility Weighted Imaging, the plaque-vessel relationship and iron accumulations in Multiple Sclerosis can be visualized, which may provide a prognostic factor of disease. Vascular imaging is a highly promising field for 7 T which is dealt with in a separate dedicated article in this special issue. The static and dynamic blood oxygenation level-dependent contrast also increases with the field strength, which significantly improves the accuracy of pre-surgical evaluation of vital brain areas before tumor removal. Improvement in acquisition and hardware technology have also resulted in an increasing number of MR spectroscopic imaging studies in patients at 7 T. More recent parallel imaging and short-TR acquisition approaches have overcome the limitations of scan time and spatial resolution, thereby allowing imaging matrix sizes of up to 128×128. The benefits of these acquisition approaches for investigation of brain tumors and Multiple Sclerosis have been shown recently. Together, these possibilities demonstrate the feasibility and advantages of conducting routine diagnostic imaging and clinical research at 7 T.

## Introduction

Three major MR vendors currently provide 7 T MR scanners for ethically approved clinical research. The number of installations in operation has increased to over 60 during the last several years. Ultra-high field MRI has been shown to supply added morphologic (Springer et al., 2016; Trattnig et al., 2015), functional (Beisteiner et al., 2011; Goncalves et al., 2015), metabolic (Bogner et al., 2011), and other biochemical (Biller et al., 2016; Zaiss et al., 2015) information about the brain.

The signal-to-noise ratio (SNR) increases with the static magnetic field strength (B_0_), which can be translated into higher spatial resolution and contrast. A recent report has shown that the image SNR may even scale supralinearly with the B_0_ (SNR~B_0_^1.65^) under certain conditions (Pohmann et al., 2016). Additional improvement in SNR (two-fold to six-fold depending on the brain region) is achieved at 7 T by efficient coil design (de Zwart et al., 2002; Wiggins et al., 2005; Wiggins et al., 2006). A volume coil is usually used for transmission to produce a relatively uniform excitation field, and phased array coils for high sensitivity detection, where each coil element is designed to optimize the SNR in a region adjacent to the coil. Application of phased arrays enables also parallel imaging (Griswold et al., 2002; Pruessmann et al., 1999), for which the SNR penalty at ultra-high fields is reduced due to onset of far-field behavior of the involved radio-frequency (RF) fields (Wiesinger et al., 2004). This allows accelerations above three-fold with a relatively small cost in SNR, which reduces the sensitivity of a given sequence to motion and can increase measurement feasibility in a clinical setting.

The most evident advantage at 7 T in neuroimaging is to gain higher spatial resolution and contrast for imaging gray and white matter disease. In the last few years, *in vitro* studies have shown new insights into the pathology of the cerebral cortex at 7 T, such as, for example, in cortical lesions in cerebrovascular disease (De Reuck et al., 2014) and Multiple Sclerosis (MS) (Yao et al., 2014), or in malformations of cortical development (MCDs), e.g., in Focal Cortical Dysplasias (FCDs) (Zucca et al., 2016) and a growing number of clinically focused *in vivo* studies have been performed at 7 T (De Ciantis et al., 2016; de Graaf et al., 2012; Harrison et al., 2015; van Veluw et al., 2013). Imaging of the hippocampus, especially its subunits, and the occasionally only subtle changes engendered by recurrent epileptic seizures, is assumed to profit from higher spatial resolution (Wisse et al., 2012).

MR neuroimaging also benefits from an increase in tissue contrast. Phase images from T_2_^*^-weighted sequences acquired at 7 T are characterized by an outstanding tissue contrast (Duyn et al., 2007), since structures with slightly different magnetic properties introduce detectable field variations at high B_0_. These field gradients are mostly prominent in the proximity of tissues with a high iron content (Haacke et al., 2005), densely distributed myelinated axons (Lodygensky et al., 2012) and veins, where the deoxyhemoglobin level is high (Reichenbach et al., 1997). In Susceptibility Weighted Imaging (SWI) valuable phase information is incorporated into magnitude imaging (Reichenbach and Haacke, 2001), using a phase mask which enhances the contrast between structures of interest. At 7 T, in the absence of a homogeneous volume reference coil (e.g. a body coil), consideration needs to be given to how phase images from the array coils are combined as the phase measured with each coil element reflects not only magnetic properties of the tissue, but also of the coil sensitivity profile, which needs to be removed. Using coil combination methods not optimized for ultra-high field applications (such as homodyne filtering (Noll et al., 1991)) may lead to the appearance of microbleeds, with potential clinical consequences (Haacke et al., 2015). Several methods for optimal coil combination in the absence of a homogenous reference coil have been proposed in recent years (Parker et al., 2014; Robinson et al., 2011; Robinson et al., 2015), the relative merits of which have been reviewed elsewhere (Robinson et al., 2016). 7 T may allow a superior assessment of the plaque-vessel relationship (Dal-Bianco et al., 2015b), iron deposits (Bagnato et al., 2011b), and temporal evolution (Absinta et al., 2013) in MS employing SWI than at lower B_0_. Also the depiction of hemorrhagic diffuse axonal injury (Moenninghoff et al., 2015), and the evaluation of the microvasculature in gliomas may be improved using SWI at 7 T (Di Ieva et al., 2013).

Because MR spectroscopy (MRS) has had to overcome several major technical challenges at 7 T (Moser et al., 2012; Posse et al., 2013), most prior studies are methodological in nature. Only recently have several papers with clinical application been published that highlight specific advantages of 7 T MRS and MR spectroscopic imaging (MRSI) for selected brain disorders, e.g., brain tumors (Heckova et al., 2016; Li et al., 2015) and MS (Srinivasan et al., 2010; Strasser et al., 2016a), by providing access to a far larger number of chemical compounds (Bogner et al., 2012; Tkáč et al., 2001).

The number of *in vivo* studies that compare 7 T with 3 T in neuroimaging is growing (Bian et al., 2014; de Graaf et al., 2013; Madai et al., 2012; Springer et al., 2016; Tallantyre et al., 2009), demonstrating concerted interest in evaluating the potential of ultra-high field (UHF) for clinical use.

The aim of this review is to provide an overview of the additional clinical benefit of 7 T MRI compared to lower B_0_ for clinical MR examinations in neuroimaging.

## Multiple sclerosis

### Cortical lesions

**Fig. 1:**
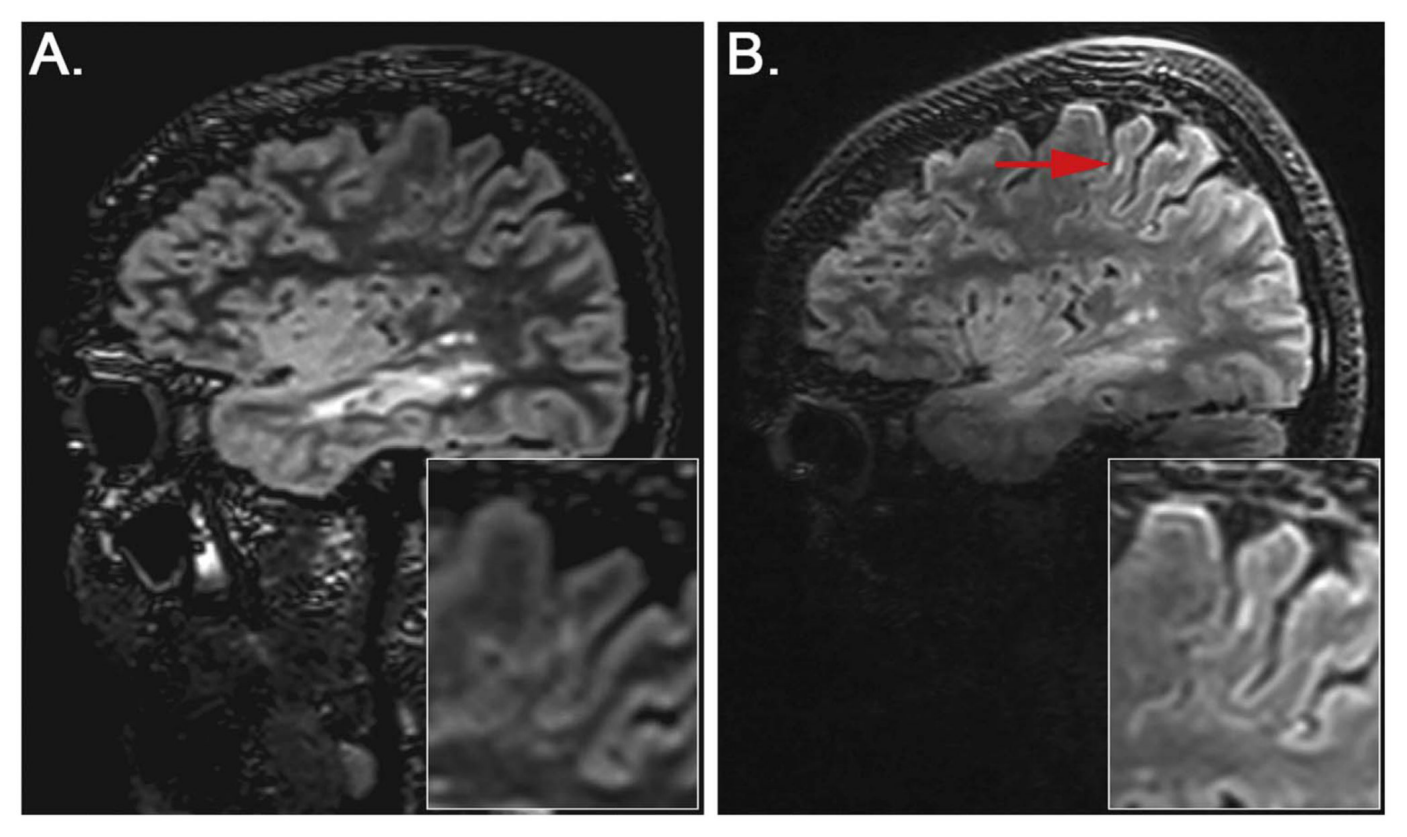
Sagittal 3D DIR of an MS patient acquired at 3 T (A) and 7 T (B). A cortical lesion - type I neocortical lesion (NL) (Yao et al., 2014) (leukocortical lesion, mixed lesion) (red arrow) - is more clearly depicted at 7 T.

The number of detected cortical lesions has been shown to be higher at 7 T, with about 30–50% of histopathologically confirmed cortical MS lesions being detected at 7 T (Van Munster et al., 2015) (Fig. 1).

In patients with MS, cortical lesions can be more precisely classified as cortically, cortically-subcortically or solely subcortically occurring due to the higher spatial resolution at 7 T in comparison to 3 T, mainly on MP2RAGE images but also using 3D DIR (Springer et al., 2016). This is consistent with a study by Tallantyre et al. (2010) in which high-resolution 7 T imaging using T1w MP-RAGE (Mugler and Brookeman, 1990) was shown to be helpful in definitively assessing the localization of lesions with respect to the cortical/subcortical boundary compared to 3 T imaging employing FLAIR, DIR, and T1w MP-RAGE. Harrison et al. found that, in MS, Expanded Disability Status Scale (EDSS) scores accorded more with cortical lesion volume than WM lesion volume (Harrison et al., 2015), supporting the clinical relevance of cortical lesions. The inversion recovery sequence FLAIR, in which the inversion time is chosen such that CSF contributes negligibly to the total signal is a highly useful sequence in cortical lesion detection at 7 T (Hajnal et al., 1992). There are, however, problems with high Specific Absorption Rate (SAR) due to the utilization of multiple 180° refocusing pulses, which usually allows only a thin brain slab to be imaged using 2D, or requires the use of thick slices. Whole brain imaging becomes feasible in 3D with application of variable flip angle readouts, i.e. SPACE (Grinstead et al., 2010). In a study by Kilsdonk et al. (2013) the highest number of cortical gray matter (GM) lesions were detected using 3D-FLAIR; 89% more than by using 3D-DIR, 87% more than when employing 2D-T2w and 224% more than using 3D-T1w. The higher number of detected GM lesions on 3D-FLAIR than on 3D-DIR was mostly due to a better detection of mixed lesions. In the patient-wise comparison the higher total GM and mixed-lesion detection at 3D-FLAIR was statistically significant; the detection of purely intracortical lesions was higher with 3D-DIR compared with the other sequences, although this difference was not statistically significant. Daams et al. (2013) concluded that future studies should focus on comparing different sequences at ultra-high field, such as DIR, T2, and FLAIR. In our experience this should be performed in combination with MP2RAGE (Marques et al., 2010), because no definite consensus exists as to the optimal sequence for cortical lesion depiction. MP2RAGE provides image quality which is superior to its predecessor MP-RAGE, because T2* and receive field (B_1_^-^) dependencies are removed by the combination of two images with different inversion times. The B_1_^-^ field becomes visibly heterogeneous at 7 T, which is observable in MP-RAGE images and can hinder correct diagnosis in brain regions affected by strong signal decay.

FLAIR images at 7 T have a hyperintense rim at the pial surface of the cerebral cortex which corresponds to the outer, glia-rich layers of the cortex (layers I – III) (van Veluw et al., 2015). Absinta et al. described the higher background T2 relaxation times of the cortex relative to white matter (WM), that are highest in the subpial cortical layers, as a reason for low cortical lesion contrast (Absinta et al., 2016a). Beneath the hyperintense signal of outer, glia-rich layers of the cortex we observe a slight decrease in the signal of the cortex and lesions – both in the cortex and WM - compared to 3 T on both 3D DIR and 3D FLAIR. Subpial lesions - the most prevalent subtype of cortical demyelination - broadly comprise the subpial layers of the cortex, and the depths of the sulci (Absinta et al., 2016a). Plaque-like subpial demyelination is a specific characteristic of MS; it is found seldom in other inflammatory and neurodegenerative CNS conditions (Fischer et al., 2013).

MRI signal changes deduced from the interruption of the cortical architecture may be used to improve subpial lesion detection (Absinta et al., 2016a; Mainero et al., 2015). Mainero et al. (2015) evaluated differences in laminar quantitative T2* maps throughout the cortical width, which at 7 T are sensitive to tissue composition at a cellular level (Zwanenburg et al., 2011). They found that patients suffering from MS showed significantly increased T2*, which was not dependent on cortical thickness, consistent with cortical myelin and iron loss, in comparison to controls. In early disease, T2* changes were focal and primarily limited to a 25% superficial depth, and also localized adjacent to cortical sulci. In later stages of the disease, T2* changes affected deeper cortical laminae, multiple cortical areas, and gyri. Their findings reveal a gradient in the existence of cortical pathology throughout the stages of MS. Hence they demonstrated *in vivo* evidence for the existence of a cortical pathological process driven from the pial surface.

In a study by Nielsen et al. (2012), more cortical, especially subpial, lesions were detected in the bilateral precentral gyri, using T2* FLASH at 7 T compared to the DIR sequence and the multi-echo MPRAGE (MEMPR) at 3 T. With 7 T T2* FLASH data considered the reference standard, the sensitivity of the DIR sequence at 3 T *in vivo* for cortical lesions was modest according to the MR in MS (MAGNIMS) consensus guidelines (Geurts et al., 2011). It was suggested by the authors that 7 T be established as the gold standard in future consensus guidelines.

Nielsen et al. (2013) also found, using T2*-weighted images at 7 T, that, in a heterogeneous MS cohort, type III-IV cortical lesions had the strongest relationship to physical disability. There was a significant correlation with four of 11 neuropsychological test variables. Also for WM lesion volume, as well as for type I cortical lesions, a significant association with six of 11 neuropsychological test variables was shown. The authors concluded that leukocortical and subpial cortical lesions detected on 7 T FLASH-T2* sequences may serve as potential biomarkers of neurologic and cognitive status in patients suffering from MS.

### Central venules

**Fig. 2:**
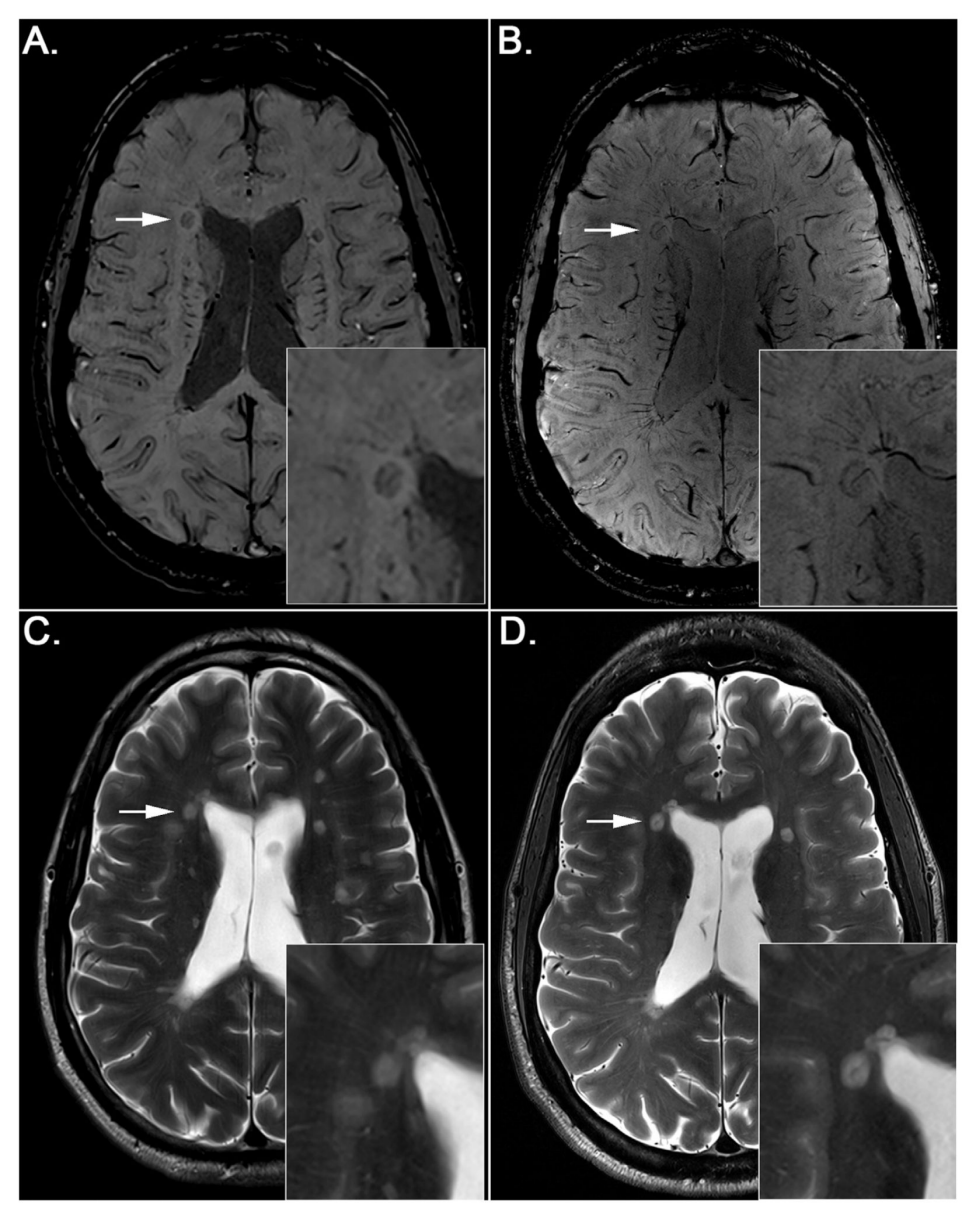
Axial SWI images of an MS patient acquired at 3 T (A) and 7 T (B) and corresponding T2-weighted images acquired at 3 T (C) and 7 T (D). Note iron deposits (rims) and central vessels within MS lesions in SWI images (A, B) (white arrows). In T2-weighted images central vessels are difficult to detect (C, D) (white arrows).

A number of authors have described central venules in MS lesions as a central feature of the disease. The phenomenon was first reported by Rindfleisch (1863) and by Charcot (1868). More recently, the perivenular distribution of MS lesions has been highlighted by high-field and ultra-high field MRI, and disease specificity has been attributed to this distribution (Kilsdonk et al., 2014b; Mistry et al., 2013; Rae-Grant et al., 2014; Tallantyre et al., 2011) (Fig. 2).

Tallantyre et al. (2009) compared 3 T and 7 T T2* imaging in 358 brain lesions identified on 3 T FLAIR images in seven patients with demyelinating brain disease with a focus on the presence or absence of a central vessel within the lesion. A central vessel could be identified in 45% of visible lesions using 3 T T2* weighted imaging, and in 87% of visible lesions at 7 T. Tallantyre et al. (2011) also addressed the question of whether it is feasible to employ T2* imaging at 7 T to distinguish between MS and asymptomatic non-MS WM brain lesions. Twenty-eight patients with known MS and a control group of 17 non-MS patients with cerebral WM lesions were examined. The location of the WM lesions was identified as an important factor for the differentiation, as in patients with MS 80% of the lesions had a perivenous appearance in comparison to just 19% in patients without MS. Using 7 T T2*-weighted MRI, a reliable discrimination between all patients with clinically definite MS (> 40% lesions perivenously localized) from those without clinical MS (< 40% lesions perivenously localized) was possible. The presence of a perivenous lesion was shown to be more predictive of MS than a subcortical or a periventricular lesion location.

Using T2* imaging at 7 T, Mistry et al. (2013) studied twenty-nine patients with brain lesions detected on clinical MRI, who were referred because of possible MS. They reported a 100% positive and negative predictive value for the diagnosis of MS, according to whether more or less than 40% of their lesions were perivenous. These results need to be validated in larger and multicenter patient cohorts in the near future.

### Iron deposits

Iron deposits in brain parenchyma may be linked to a multiplicity of pathological processes in patients with MS and might potentially be used as an *in vivo* MRI marker of disease pathology. Brain tissue iron content was assessed by Bagnato et al. (2011a) in two patients with MS. The authors employed a 3D multi-echo gradient echo sequence to reconstruct R2* maps and correlate the tissue iron content with histological analyses. An increase in R2* was described as being highly sensitive for the detection of iron depositions although signal alterations were not always associated with iron in the histological analyses, which was tentatively attributed to iron loss in sample preparation. The authors concluded that the identification of iron should be evaluated in combination with additional topographical and clinical information. The sources of iron identified included oligodendrocytes in normal-appearing white matter (NAWM) and activated microglia / macro-phages at the borders of WM lesions, as well as iron precipitation in aggregates typical of microbleeds in WM lesions. This is in accord with Hametner et al. (2013), who reported that iron was apparently released from dying oligodendrocytes in active MS lesions, leading to an extracellular accumulation of iron and an uptake into macrophages and microglia. The latter showed signs of cell degeneration. Furthermore, the authors noted that iron accumulated in astrocytes and axons at lesion edges and within the centers of lesions.

Both Absintha et al. and Hammond et al. proposed that the peripheral paramagnetic rim (Absinta et al., 2013, 2016b; Dal-Bianco et al., 2015a; Hagemeier et al., 2012; Hammond et al., 2008; Yao et al., 2012) that can be perceived on T2*- magnitude and susceptibility-weighted phase images (Fig. 2) is a potential marker of chronic active lesions (Absinta et al., 2016b; Hammond et al., 2008). The studies performed to date are in complete concordance about the extent to which the MR magnetic susceptibility signal change in the lesion rim may be caused by iron deposits (iron-laden macrophages, ferritin, or hemosiderin deposits) (Bagnato et al., 2011a; Pitt et al., 2010; Walsh et al., 2013; Yao et al., 2012) or by oxidative stress and rupture of the microarchitecture of the parenchyma, including demyelination.

Absinta et al. (2013) showed that when combining dynamic contrast-enhanced MRI with T2* imaging, particularly phase data at 7 T, the front of demyelination and inflammation in MS lesions may be identified. They demonstrated that in centripetally enhancing lesions, the paramagnetic phase rim reflects the initial opening of the blood brain barrier at the inflammatory lesion edge. In a more recent study at 7 T, Absinta et al. (2016b) examined centripetally and centrifugally enhancing lesions in 17 patients with MS. While in centripetal lesions, a phase rim appeared simultaneously with initial contrast enhancement, no centrifugal lesions showed phase rims at any point in time. The phase rim in 12 of 22 centripetal lesions persisted after enhancement resolved. The centripetal lesions with a persistent rim had less volume shrinkage and became more T1 hypointense between 3 and 12 months in comparison to the centripetal lesions with a transient rim. Persistent rims pathologically corresponded to iron-laden inflammatory myeloid cells at the edge of chronic demyelinated lesions.

With respect to MS specificity, paramagnetic rims have not been observed in vascular lesions (Kilsdonk et al., 2014b), and are seldom present in Susac syndrome (Wuerfel et al., 2012). They may be observed in infectious and neoplastic disorders (Absinta et al., 2016a). Sinnecker et al. (2015) investigated a rare case of the simultaneous presentation of natalizumab-associated Progressive Multifocal Leukoencephalopathy (PML) and ongoing MS activity and found that ultra-high-field MRI is able to differentiate between early PML and MS lesions. Whereas a central vein was visible within ringenhancing MS plaques on 7 T T2* imaging, punctate or milky way-like T2 lesions were apparent before the development of confluent PML lesions. In another study Sinnecker et al. (2012) observed that it was possible to distinguish Neuromyelitis Optica Spectrum Disorders (NMOSDs) from MS at 7 T, where the protocol incorporated T2*-weighted FLASH and FLAIR sequences. They compared 10 patients with NMOSDs with 18 patients suffering from MS. While a central vein was prevalently visible within MS plaques (92%) and a characteristic hypointense rim (23%), the appearance of WM changes in NMOSDs was nonspecific; they were sporadically neighbored by a blood vessel (35%) and a hypointense rim was identified seldomly (2%). Cortical pathology was not present in patients with NMOSDs.

Several groups have used a fusion of a FLAIR sequence and a blood-sensitive sequence, e.g., SWI (Grabner et al., 2011) or T2* imaging, to examine the relation of intraplaque veins and plaque development in MS over time (Dal-Bianco et al., 2015b), and to facilitate the differentiation of MS and microangiopathic lesions at 7 T (Kilsdonk et al., 2014b). Kilsdonk et al. correlated three morphological properties of MS lesions (the presence of a central vessel within lesions; the presence of hypointense rims around MS lesions; the presence of hypointense lesions on T2*) with the patient's clinical characteristics, but found that these properties did not show a relationship to clinical characteristics or MS disease subtypes (Kilsdonk et al., 2014a). Dal-Bianco et al. described a relationship between veins and plaque development in MS at 7 T, deducing that increased apparent venous calibers in early MS plaques may be due to increased venous diameters or increased oxygen consumption (Dal-Bianco et al., 2015b).

## Cerebrovascular disease

Cortical microinfarcts (CMIs) are small infarcts (between 50 μm and a few mm) that usually remain undetected with conventional MRI fieldstrengths or at autopsy (Smith et al., 2012; van Veluw et al., 2013). Van Veluw et al. (2013) demonstrated the potential of ultra-high-field MRI to detect *in vivo* cortical microinfarcts. Cortical cerebral micro-infarctions were studied by Van Veluw et al. employing 3D FLAIR, 3D T2w Turbo Spin Echo (TSE) and 3D T1w at 7 T in 22 elderly subjects, and in 15 formalin-fixed coronal brain slices of six subjects with Alzheimer's disease and vascular pathology. In the latter group six lesions, with MRI morphology similar to the 15 cortical lesions of the first group, were correlated to histopathology, and of these six cortical lesions five were verified as cortical microinfarcts.

A study by De Reuck et al. (2014) demonstrated that the detection of different types of CMIs is enabled by 7 T MRI. They used postmortem 7 T MRI in comparison with the pathological examination to study CMI in 157 brains of patients with dementia – amongst them 18 with vascular disease and 12 with Alzheimers disease (AD) associated with cerebral amyloid angiopathy (AD-CAA). Significantly more CMIs were detected in patients with vascular disease and AD-CAA. The mean number of CMIs detected by 7 T MRI and by neuropathological examination were comparable.

In another study, more CMIs were detected in AD patients than in control subjects. The number of CMIs corresponded to global cognitive performance and their presence was primarily attributed to AD rather than to Cerebral Amyloid Angiopathy (CAA) (van Rooden et al., 2014).

In cerebrovascular disease, cortical lesions can be more precisely classified as cortically, cortically-subcortically or solely subcortically occurring due to the higher special resolution at 7 T in comparison to 3 T, mainly on MP2RAGE images, but also using 3D DIR (Springer et al., 2016).

An overview of 7 T imaging of Small Vessel Disease (SVD) was presented in the work of Benjamin et al. (2015). Madai et al., (2012) in their study on ischemic stroke, which encompassed lacunar strokes, demonstrated that the internal structure of stroke lesions was delineated with higher detail on the 3D MPRAGE at 7 T in comparison to 3 T. Improved image contrast at 7 T between lesions and healthy tissue enabled better depiction of periinfarct alterations.

Potter et al. (2015) suggested that enlarged perivascular spaces are an additional MRI marker for SVD. In their study they were associated with age, lacunar stroke, and white matter lesions. Using 7 T MRI, the depiction of perivascular spaces was improved and they could be visualized in greater detail than at 3 T (Madai et al., 2012). Another study reported that the level of enlargement of juxtacortical perivascular spaces was significantly related to CAA severity; hence, enlarged juxtacortical perivascular spaces may be a potential marker for the severity of CAA (van Veluw et al., 2016).

## Microbleeds

Conijn et al. (2011) described findings showing that in comparison to a 3D T2*-weighted sequence at 1.5 T, the presence and number of detected cerebral microbleeds (CMB) were increased using a 3D dual-echo T2*-weighted sequence at 7 T. Furthermore, that the reliability of the depiction of CMBs improved. Bian et al. (2014) demonstrated that SWI at 7 T was more sensitive for depicting CMBs in subjects treated with radiotherapy than SWI at 3 T, when the localization of the CMBs was not in regions with prominent susceptibility artifacts, which occur due to stronger signal dephasing in regions with substantial B_0_ inhomogeneities at 7 T. Theysohn et al. (2011), employing high-resolution SWI with an excellent phase contrast at 7 T, could depict 129 CMBs, in comparison to 75 at 1.5 T when employing clinical SWI; when applying a T2* imaging sequence they could depict 101 CMBs at 7 T and 33 at 1.5 T.

Moenninghoff et al. (2015) showed that for the detection of hemorrhagic diffuse axonal injury 7 T SWI was more sensitive in comparison to 3 T SWI, a statistically significant finding.

## Mesial Temporal Lobe Epilepsy (MTLE)

### Mesial Temporal Sclerosis (MTS)

**Fig. 3:**
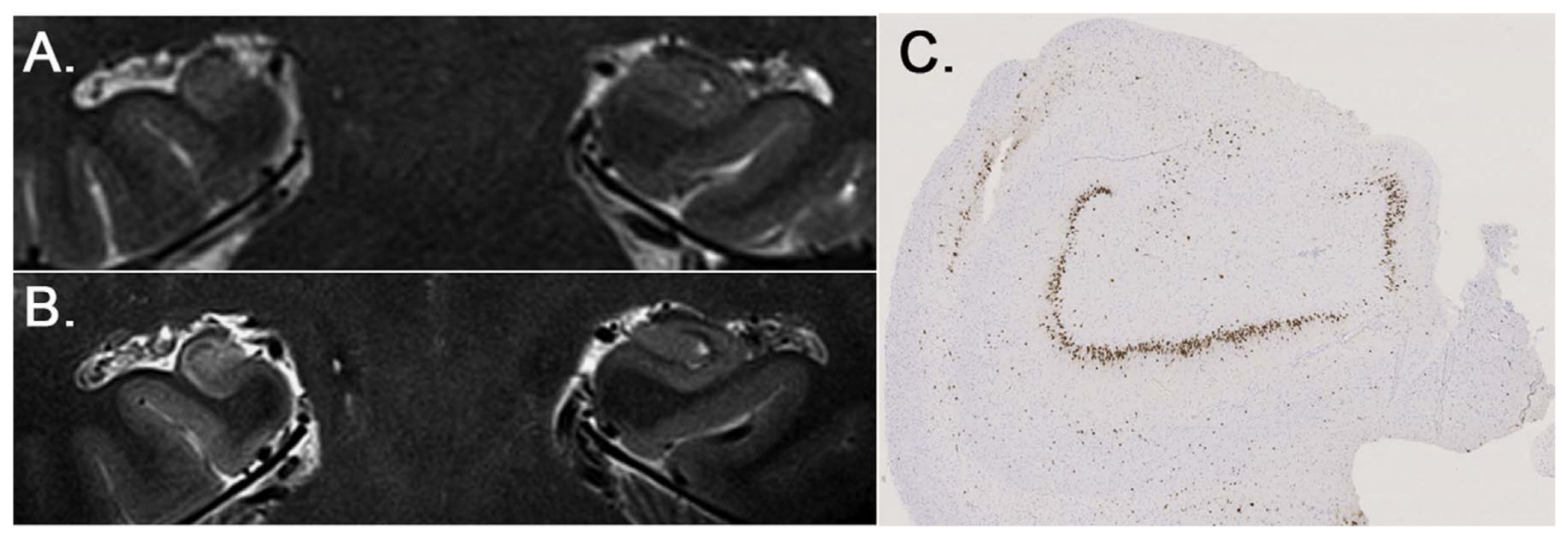
Coronal T2-weighted images at 3 T (A) and 7 T (B) of a patient suffering from right-sided MTS type 1. Neuronal loss in all hippocampal subfields CA1, CA2, CA3, and CA4. Corresponding histopathological correlate, NeuN staining (C).

In Mesial Temporal Lobe Epilepsy (MTLE), a higher diagnostic confidence for the diagnosis and exclusion of Mesial Temporal Sclerosis (MTS) including which subfields were affected was demonstrated at 7 T compared to 3 T. (Springer et al., 2016) (Fig. 3). This was primarily attributable to the higher spatial resolution at 7 T, specifically in the coronal T2 weighted TSE sequence. Even though the hippocampus is quite close to the skull base, TSE sequences are quite robust to susceptibility artifacts in that area. Additionally, TSE at 7 T is an excellent choice in discrimination between different tissue types because of the increased T1 and decreased T2 relaxation times.

In-plane spatial resolutions of up to 100 μm (Prudent et al., 2010) and a slice thickness of between 0.7 and 3 mm, may be obtained *in vivo* using T2-weighted imaging (Theysohn et al., 2009; Wisse et al., 2012). Wisse et al. (2012) demonstrated that it is feasible to delineate the main subunits of the hippocampus along its length at 7 T *in vivo*.

Patients suffering from Temporal Lobe Epilepsy (TLE) in clinical practice are preoperatively evaluated, including imaging of the mesial temporal lobe at 1.5 or 3 T, where the superiority of 3 T compared to 1.5 T for volumetry and morphological details has already been shown in recent studies (Chakeres et al., 2005; Theysohn et al., 2009).

Sequence protocols at 7 T typically enable a morphological delineation of the internal hippocampal structures in detail due to the higher spatial resolution (Breyer et al., 2010). Coras et al. (2014) employed T2 weighted sequences and Diffusion Tensor Imaging (DTI) at 7 T to investigate 15 nonsclerotic and 18 sclerotic hippocampi *ex vivo*, and images were correlated to histology. Differences in signal intensity were attributed to seven unequivocally discernable layers and to anatomic borders in the case of nonsclerotic hippocampi. A significant loss of volume and an increase in signal intensity along the pyramidal cell layer was revealed in all sclerotic hippocampi. An assignment of the increase in signal intensity on T2 weighted images to a definite histopathologic correlate such as decreased neuronal or increased glial cell density was not possible, although intrahippocampal fiber tracts and projections were distorted in samples of hippocampal sclerosis, indicating a complex disorganization of the extracellular matrix, fiber networks, as well as the cellular composition.

To overcome motion artifacts, Marrakchi-Kacem et al. (2016) recently introduced an acquisition protocol for imaging the hippocampal structure at 7 T. The full slab containing the hippocampus was divided into separate slabs, each of them requiring a lower acquisition time. The acquired single slabs were combined within a high-resolution, 3D-consistent slab, using a robust registration approach. They found this method to be effective in decreasing acquisition time and hence motion artifacts when performing ultra-high resolution imaging of the hippocampus.

Wisse et al. (2016) at 7 T MRI recently investigated the feasibility of automatically segmenting hippocampal subunits and the entorhinal cortex instead of performing a labour intensive manual segmentation, and described a high accuracy for most subunits.

The development of an online computational anatomical atlas of the hippocampus has been reported by Yushkevich et al. (2009). The atlas was derived from postmortem specimens scanned with high-resolution 9.4 T MRI, to support subfield segmentation in hippocampus imaging and image analysis. In their recent work (Yushkevich et al., 2015), the variability in the existing manual segmentation protocols to identify hippocampal and parahippocampal substructures in MRI, qualitatively as well as quantitatively, is described, to guide the development of a harmonized substructure segmentation protocol.

### Malformations of Cortical Development (MCD)

Higher resolution and improved gray/white matter contrast in the MP2RAGE sequence at 7 T (O'Brien et al., 2014) enables better gray/white matter differentiation. This proved valuable in the exclusion or diagnosis of MCD, a common cause of drug-resistant epilepsy (Barkovich et al., 2012), in a recent study by our group (Springer et al., 2016). The difference between 3 T and 7 T was not statistically significant, however.

Some imaging signs of focal cortical dysplasias (FCD) (Blumcke and Muhlebner, 2011) are often difficult to depict or to exclude. These include an ill-defined, thickened cortex, or blurring of the gray-white matter junction. These findings have to be confirmed with multiplanar imaging or isovoxel reconstructions to exclude volume averaging, and could benefit from the higher spatial resolution provided by 7 T. On 7 T T2* FLASH images, a disruption of low-signal subcortical U fibers in the area of dysplasia was perceived (Madan and Grant, 2009). The lesion described was depicted at both field strengths on the MP-RAGE and axial T2 TSE images, but in the future it is hoped that the improved signal, contrast, and resolution of 7 T will help to depict more subtle cortical dysplasias. Zucca et al. (2016) studied the correlations between *ex vivo* 7 T imaging and histological and ultrastructural patterns of type II FCD in 13 patients. On T2 weighted imaging a quantitative region of interest (ROI) based analysis was performed to evidence the presence and the border of type II cortical dysplasia. The quantitative approach may help to differentiate the lesions and perilesional areas, also in MRI-negative cases.

In a study by De Ciantis et al. (2016), 7 T MRI detected structural abnormalities not previously depicted at lower field strengths in six (29%) out of 21 patients with focal epilepsy. The authors used a dedicated protocol that included 3D T1w fast-spoiled gradient echo (FSPGR), 3D susceptibility-weighted angiography (SWAN), 3D magnetization-prepared (MP)-FLAIR, 2D T2*-weighted targeted dual-echo Gradient-Recalled Echo (GRE), 2D T2W TSE, 2D gray-white matter tissue border enhancement (TBE) TSE- Inversion Recovery (IR) (Costagli et al., 2014). The added value of 7 T in detecting structural lesions was achieved using GRE, FLAIR, or both.

De Ciantis et al. also studied the potential of 7 T *in vivo* in imaging polymicrogyria. In 10 adult patients suffering from polymicrogyria, diagnosed before using 3 T MR imaging, they examined whether additional characteristics might be revealed. Additional sequences at ultra-high field comprised 3D T2* susceptibility-weighted angiography as well as 2D TBE TSE-IR. At 7 T, perisylvian polymicrogyria was diagnosed to be bilateral in six patients, unilateral in three, and diffuse in one. Of the six bilateral abnormalities four had been assessed as unilateral at 3 T.

### Cerebral Cavernous Malformations (CCMs)

Another possible cause for seizures are Cerebral Cavernous Malformations (CCMs), for which MRI has been established as the gold standard for diagnosis. In a recent study by Frischer et al., CCMs and possibly associated Developmental Venous Anomalies (DVA), were assessed in 24 patients on the SWI sequence at 7 in comparison to 3 T (Frischer et al., 2012). Histopathology was available in 11 of 24 patients in surgically resected lesions to prove the diagnosis of CCMs and potentially associated DVAs. Significantly more CCMs were identified on 7 T SWI than on 3 T SWI and a higher number of associated DVAs were identified at 7 T compared to 3 T. Their detection is relevant, since CCMs with associated DVAs have a higher risk for clinically significant hemorrhages.

Schlamann et al. (2010) examined ten patients with known cavernomas using T2*-weighted GRE sequences at 1.5 T and at 7 T. In one patient, an additional hypointensity was depicted at 7 T, which was not visible at 1.5 T, even retrospectively. A better visualisation of small cavernomas may help to explain cryptogenic seizures.

## Brain tumors

For imaging brain tumors, attempts have been made to rely on non-contrast-enhanced techniques. SWI at 7 T is used in brain tumors to explore the intratumoral structure in gliomas, in particular with regard to neoangiogenesis and microhemorrhage. The formation of pathological microvessels is a marker for a malignant transformation when low-grade gliomas (World Health Organization (WHO) grades I and II) show a progression to high-grade gliomas (WHO grades III and IV). In addition, the oxygen extraction within a brain tumor may increase due to elevated metabolism within the lesion, with higher deoxyhemoglobin levels leading to more pronounced veins on SWI. Grabner et al. observed that 7 T SWI appears to be capable of tracking changes in brain tumor vasculature resulting from treatment with antiangiogenic drugs (Grabner et al., 2011). Di Ieva et al. (2013) in 36 patients with brain gliomas (grades II–IV) scanned at 7 T applied computer-aided fractal image analysis. Higher fractal dimension values, as a measure of geometric complexity of intratumoral SWI patterns, were found in groups with a higher histologic grade. A statitistically significant difference was observed between histopathological groups, suggesting that this approach may provide a morphometric imaging biomarker of tumor grade. A more exact tumor grading with regard to localization of areas of dedifferentiated cells could help in guiding biopsy.

A high resolution 7 T MRI protocol with a 0.8 mm isotropic voxelsize has been proposed by de Rotte et al. (2014) for imaging the pituitary gland. The feasibility of this was demonstrated in healthy control subjects and patients with Cushing's disease. A second study of patients with Cushing's disease by de Rotte et al. (2016) reported good interobserver agreement of image assessment for diagnosis and exclusion of pituitary microadenomas both for 7 T and 1.5 T MRI, while lesions were detected more accurately with 7 T MRI.

## Clinical fMRI

The primary clinical use of functional Magnetic Resonance Imaging (fMRI) is in presurgical planning for patients with brain tumors and epilepsy. Eloquent brain regions close to pathologies are mapped with fMRI using a relevant task (Duffau, 2006), and regions defined as active are spared in surgery to ensure that vital function is preserved. In practice, integrating fMRI results into the treatment plan leads to more aggressive resection and decreased surgical time (Petrella et al., 2006). The opportunity to increase the reliability of localizations or reduce the scan time using 7 T is attractive, particularly as many patients find it hard to perform tasks and remain still for prolonged periods. The relatively limited number of 7 T installations at hospital sites and the fact that the current generation of 7 T MRI systems are not certified for clinical use has meant that, to date, only a small number of studies have been conducted on the feasibility and potential advantages of performing clinical fMRI at 7 T.

In a study of 17 patients referred for presurgical localization of the primary motor hand area, in which localizations were performed at both 3 T and 7 T, Beisteiner et al. (2011) showed a clinically relevant increase in functional sensitivity at the higher field strength. A similar study of language function yielded a more nuanced result, with sensitivity being higher at 7 T in Wernicke's but not Broca's area, as a result of increased susceptibility-related signal loss and Nyquist ghost artefacts in the UHF measurements in the Broca region (Geissler et al., 2014).

SNR increases with field strength, but the sensitivity to blood oxygenation level dependent (BOLD) signal changes in fMRI is proportional not to image SNR but to time-series SNR (tSNR). The thermal noise contribution to tSNR is quite constant with field strength whereas the noise arising from motion and physiological processes both scale with image intensity, and thereby, field strength (Kruger and Glover, 2001). Thermal noise dominates at high resolution, so BOLD sensitivity gains with field are most significant in that regime (Triantafyllou et al., 2005). In presurgical planning, SNR gains achieved with improved RF coils and higher field have largely been invested in improving the quality of results (Beisteiner, 2013) rather than reducing scanning time or resolution. Imaging resolution has, in fact, remaining broadly unchanged since early clinical fMRI work (see, e.g. circa 2×2×3 mm^3^ voxels in (Beisteiner et al., 2000)). This wide-spread use of “standard” resolution in presurgical planning explains the relatively modest improvement in sensitivity with field observed in the two comprehensive 3 T - 7 T comparison studies carried out to date (Beisteiner et al., 2011; Geissler et al., 2014).

A shift of resection margins by only 1 mm close to eloquent brain areas may determine whether post-operative deficits are reversible or permanent (Haglund et al., 1994). Susceptibility-related distortions scale with field strength, so it is particularly important at UHF that these be corrected. This can be done using a field map acquired either prior to, or after, the fMRI runs (Jezzard and Balaban, 1995; Robinson and Jovicich, 2011). This ‘static’ approach captures B_0_ at a single point in time, but is subject to error where there are significant changes in B_0_ due to breathing or motion. In ‘dynamic’ field mapping, in contrast, a map of B_0_ is calculated from the phase of the Echo Planar Imaging (EPI) volumes in the fMRI time series themselves. This requires the use of multi-echo EPI (Hutton et al., 2002; Visser et al., 2012; Weiskopf et al., 2005), ‘jittering’ of the echo time between different values for odd and even time points (Dymerska et al., 2015) or the use of a short prescan which can be used to determine the phase offset of each RF coil in the array so that the phase from each image in an unmodified single-echo EPI sequence can be converted to a field map (Dymerska et al., 2016). Correcting distortions has been shown to be clinically relevant to decision-making in presurgical planning at 7 T (Dymerska et al., 2014).

Head motion is a particular problem in clinical fMRI. Patients tend to move more than healthy subjects and many of the tasks used in presurgical planning – elbow flexion, plantar flexion, overt speech - evoke head motion. Effective and comfortable head fixation is required (Edward et al., 2000), as are imaging methods which are less sensitive to motion. In accelerated EPI, for instance, modified schemes for the acquisition of the GRAPPA reference lines (Griswold et al., 2002) have recently been proposed which are more robust to motion and respiration: If the reference lines are acquired in segmented fashion, all the segments can be acquired for each slice in rapid succession, with low flip angle (Polimeni et al., 2015), replacing the conventional ‘consecu-tive slices‘ scheme by ‘consecutive segments’ which reduce motion and phase changes between segments. Alternatively, reference lines can be acquired with FLASH (Talagala et al., 2016). Both approaches lead to an increase in tSNR in tasks involving motion at 7 T (Cardoso et al., 2016a). In-plane acceleration allows a reduction of TE that is generally necessary at UHF. The TR can be likewise reduced using simultaneous multi-slice acquisition (Setsompop et al., 2012) which allows a reduction in TR by the multiband factor, increasing the SNR per unit time with only a modest g-factor penalty (Barth et al., 2016).

Delayed reactions to task cues and modified hemodynamics close to brain tumors (Fujiwara et al., 2004; Zaca et al., 2014) lead to consistent deviations from expected response timing in the clinical context. These have motivated the use of exploratory data analysis methods such as Independent Component Analysis (ICA) (Beckmann, 2012), risk mapping (Beisteiner et al., 2000) and the use of signal changes in one run as a predictor for the response in other runs (‘UNBIASED’) (Cardoso et al., 2016b). ICA has been shown to be effective in separating activation from motion artefacts in 7 T clinical fMRI (Robinson et al., 2013), while UNBIASED was able to detect signal changes which were consistent and time-locked to the task, but which were delayed or transient (Cardoso et al., 2016b). Resting-state acquisitions are increasingly being explored as means to map functional networks in patients who may have difficulty performing tasks (Kamran et al., 2014), although to our knowledge, this has not yet been applied at UHF.

**Fig. 4:**
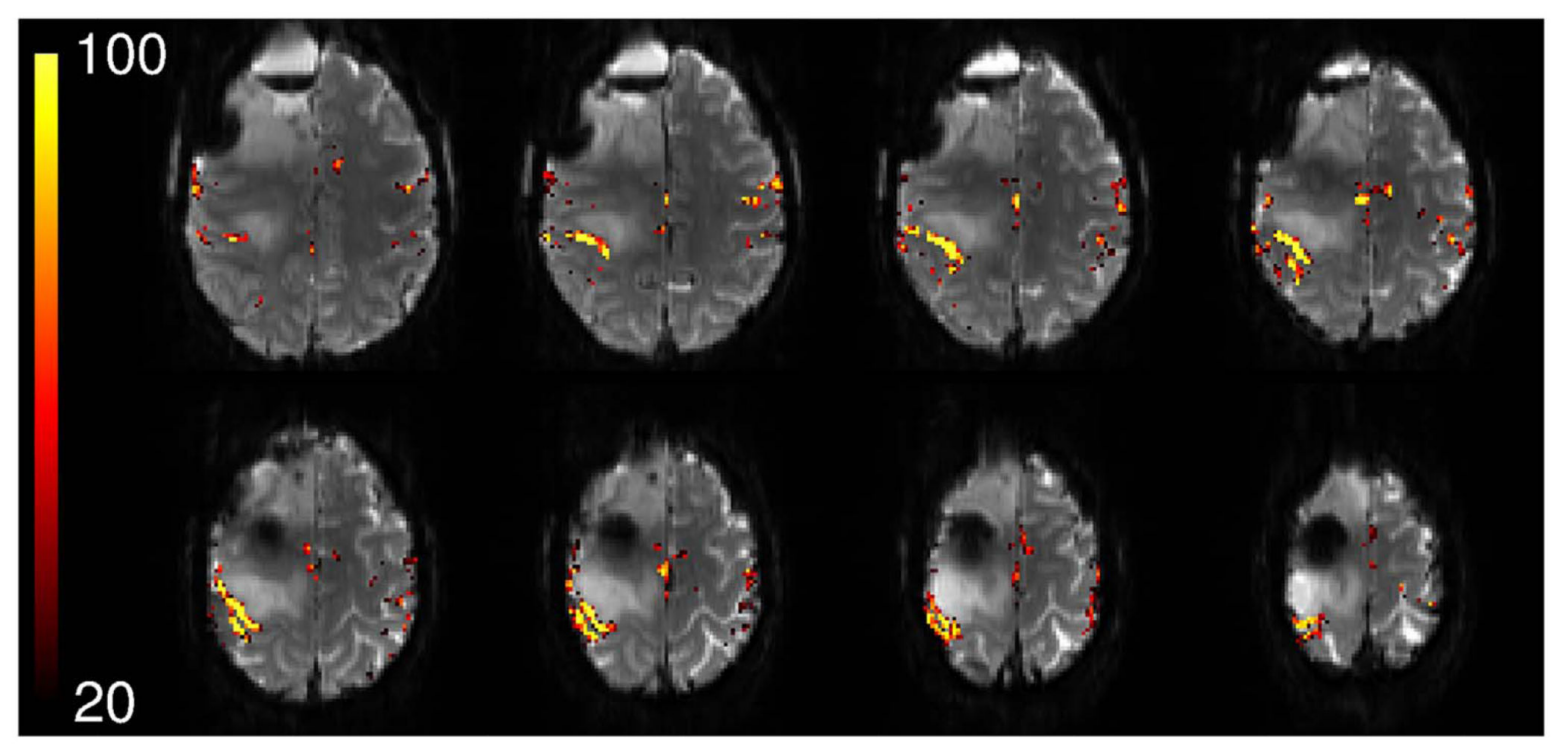
Presurgical mapping of the primary hand motor representation in a patient with 2 oligo-dendrogliomas grade II (1 right frontal, 1 right central). Activation in primary motor, somatosensory and supplementary areas can be seen in fMRI results showing voxels which were activated with between 20% and 100% reliability (overlaid on the mean EPI). No smoothing or coregistration of the functional results was performed.

Ultra-high field fMRI with high spatial and temporal resolution opens up the possibility to study more subtle disruption and reorganization of function, and allows weak activation to be detected in previously undiagnosable patients with large and rapidly evolving tumors and complex pathologies. In the example in Fig. 4, the reliability of the response in a hand task, calculated with UNBIASED (Cardoso et al., 2016b), is shown for a patient with 2 oligo-dendrogliomas grade II (1 right frontal, 1 right central). These were calculated using 10 short fMRI runs with identical timing which were dynamically corrected for distortion (Dymerska et al., 2016) and are overlaid on a temporal mean distortion-corrected EPI image.

Simultaneous multi-slice acquisitions with more motion-robust inplane acceleration are expected to be a key element in achieving the SNR per unit time required to allow the clinical fMRI community to use higher resolution acquisitions, where the tSNR benefits of UHF are most evident. This is expected to allow UHF clinical fMRI to increasingly supplant invasive diagnostic procedures such as intraoperative cortical stimulation.

## Arterial spin labeling (ASL) at 7 T

The longer T_1_ of blood (Rooney et al., 2007) and increased SNR at 7 T provide ostensibly attractive conditions for ASL, but B_0_ and B_1_ inhomogeneities make it hard to achieve a well-defined labelling slab and high labelling efficiency within SAR limits. Compared with birdcage transmit coils, dedicated neck labelling coils provide efficient, low-SAR excitation of blood in the carotids, but SAR calculations need to consider contributions from both the birdcage and neck coils (Wang et al., 2008), and such systems are not yet broadly available. Low-SAR labelling schemes such as flow-sensitive alternating inversion recovery (FAIR) (Kim, 1995) and Turbo-FLASH (Zuo et al., 2013) have been used successfully at 7 T for both pseudocontinuous ASL (pCASL) and pulsed ASL (PASL), while Gharig et al. (2012) found high-permittivity dielectric pads effective in compensating for lower B1 efficiency. FAIR labelling has been combined with fast simultaneous multi-slice readout to increased brain coverage for a particular imaging period, with low SNR penalty (Ivanov et al., 2016). These methodological inroads are expected to make first clinical ASL studies viable in the near future.

## MR spectroscopy

While MR spectroscopy (MRS) of animals, such as rats, at 7 T can be dated back to the 1990 s, the feasibility of *in vivo* MRS in the human brain at 7 T was first shown in 2001 (Tkáč et al., 2001). Since this date, despite considerable research, only few clinical applications were published until 2010. These numbers increased only recently, which is mainly related to the technical challenges associated with MRS, and particularly, with MRS imaging (MRSI) at ≥7 T (Moser et al., 2012; Posse et al., 2013). While common MRI methods benefit from increased SNR only, MRS also gains spectral resolution. This significantly improves both the number and the accuracy of detectable brain metabolites (Bogner et al., 2012; Tkáč et al., 2001), thereby making possible entirely new clinical applications.

### Single-voxel MRS at 7 T

Most early 7 T MRS patient studies were restricted to single-voxel MRS. For example, MRS in the caudate nucleus of Huntington's disease (HD) patients showed lower NAA and lower creatine concentrations than in controls (van den Bogaard et al., 2011). In the putamen, manifest HD patients show the aforementioned changes, as well as lower glutamate. In the premanifest gene carriers, absolute values of NAA, creatine, and glutamate were lower, although no significant differences were seen compared to healthy controls. In early-manifesting HD the lower concentrations of NAA and creatine in the caudate nucleus and putamen may indicate loss of neuronal integrity and impaired energy metabolism. Changes in glutamate may underpin the hypothesis of excitotoxicity of neurons (van den Bogaard et al., 2011).

MRS also provides excellent biomarkers for disease progression, as found in a longitudinal study (van den Bogaard et al., 2014). While van den Bogaard et al. (2014) reported a significant longitudinal decrease of tNAA in the putamen of all subjects (three manifest and 10 premanifest HD gene carriers), in premanifest HD converters they observed an elevated rate of tNAA decline in comparison to non-converting premanifest HD, which however was not a statistically significant effect.

Lower cerebellar NAA/Cr ratios were detected in patients with hemiplegic migraine compared to control subjects (Zielman et al., 2014). The reduced tNAA/tCre ratio in the cerebellum could be used a potential early biomarker for neuronal loss or/and dysfunction (Zielman et al., 2014).

### Neurotransmitters - GABA, glutamate, glutamine

A particular improvement at 7 T was found in the detection of the inhibitory neurotransmitter GABA, which is of low abundance and therefore difficult to quantify at lower B_0_. Investigation of GABA, Glu, and Glutamine (Gln) in different subregions of the anterior cingulate cortex (ACC) showed different relative concentration patterns (Dou et al., 2013). Dou et al. were able to show that in the pregenual (pg) ACC elevated excitatory Glu and inhibitory GABA meet a local segregation, in accordance with recently demonstrated receptor-architectonic distribution of GABA_B_ receptors in ACC (Palomero-Gallagher et al., 2009), whereas Gln distribution followed an AMPA receptor pattern (Dou et al., 2013).

An increase of GABA was found in the pons and putamen in Parkinson's disease (Emir et al., 2012). GABA measurements in the hypothalamus and occipital cortex showed a trend toward a decrease in GABA in the hypothalamus during hypoglycemia (Moheet et al., 2014). GABA and glutamate were also altered in schizophrenia patients compared to controls (Brandt et al., 2016; Marsman et al., 2014), and a GABAergic dysfunction was found within the corticostriatal pathways in children with primary complex motor stereotypes (Harris et al., 2016).

Gabapentin, used for the treatment of neuropathic pain and seizures, has been demonstrated to increase GABA concentration in the visual cortex of rodents and humans (Cai et al., 2012). Administration of gabapentin was associated with an average rise of GABA concentrations of 55.7% (6.9–91.0%), though GABA concentration changes were small within-day (average 5.6%) and also between-day (average 4.8%). A change in GABA levels, secondary to administration of the drug, was correlated inversely to the GABA level of the individual at baseline (R^2^=0.72). Further, the thalamic GABA/NAA ratio was found to increase for well-controlled epilepsy and decrease for poorly controlled epilepsy (Pan et al., 2013b).

### Onco-markers

Single-voxel 7 T MRS also offered enhanced sensitivity and specificity for quantification of the onco-marker 2-hydroxyglutarate (2-HG) and associated metabolites, with implications for better monitoring of the response of tumor patients to therapy (Emir et al., 2016). Initial clinical results have also been obtained at 9.4 T, highlighting the additional benefits at further increased B_0_. This includes the direct detection of important onco-markers such as 2-HG, which allows IDH1/2-mutations to be identified non-invasively and without complicated editing methods that lower the detection sensitivity (Bisdas et al., 2016). The first glioma patient results using ^31^P-MRSI have also been reported at 9.4 T (Mirkes et al., 2016).

### MR Spectroscopic imaging at 7 T - current and future directions

The number of MRSI studies in patients are also increasing at 7 T and higher field strengths, a fact which can partly be attributed to the continuous improvement of MRSI acquisition and hardware technology (Fillmer et al., 2015; Hangel et al., 2015; Pan et al., 2012; Strasser et al., 2013). However, most of these studies are still limited to low matrix sizes (i.e., ≤24×24) due to long measurement times (Hetherington et al., 2014; Li et al., 2015; Pan et al., 2013a; Ratai et al., 2008; Srinivasan et al., 2010).

### Recent developments in MRSI

More recent acquisition approaches have overcome the limitations of scan time and spatial resolution, allowing matrix sizes up to 128×128 (Hangel et al., 2016). The benefits for the investigation of brain tumors and MS have been shown recently with nominal in-plane resolutions as low as 2–3 mm (i.e., a 64×64 to 100×100 matrix) (Strasser et al., 2016a). Even at such high spatial resolution, single- and multi-slice ultra-short TE MRSI sequences at 7 T can image up to nine important brain metabolites (Povazan et al., 2015) in a clinically feasible scan time of ~6–7 min (Hangel et al., 2015; Strasser et al., 2016b). Apart from resolution improvements, also low-abundant J-coupled brain metabolites can be mapped at UHF, which has not been feasible previously (Heckova et al., 2016).

### Clinical applications of MRSI

The assessment of lower glutathione (GSH) levels in lesions and the GM of MS patients has become feasible for the first time, using spectral-edited MRSI at 7 T (Srinivasan et al., 2010). Recent data suggest that metabolic alterations in NAA, GSH, and myo-Inositol occur even beyond T_2_-visible MS lesions, and may thus predict the location of lesion formation (Srinivasan et al., 2010; Strasser et al., 2016a).

Using MRSI, a decrease of NAA/Cho and NAA/Cr in the hippocampi of mild traumatic brain injury patients could be detected (Hetherington et al., 2014). Abnormalities in NAA/Cr ratios have been successfully correlated to the outcome of surgical resection in epilepsy (Pan et al., 2013a).

MRSI in adult X-linked adrenoleukodystrophy has shown changes in metabolic ratios, such as lower NAA/Cr between different phenotypes and controls (Ratai et al., 2008).

Short-TE 3D-MRSI was able to provide a significantly more comprehensive neurochemical investigation of glioma and infiltrated areas at 7 T (Li et al., 2015) compared to lower field-strengths.

**Fig. 5:**
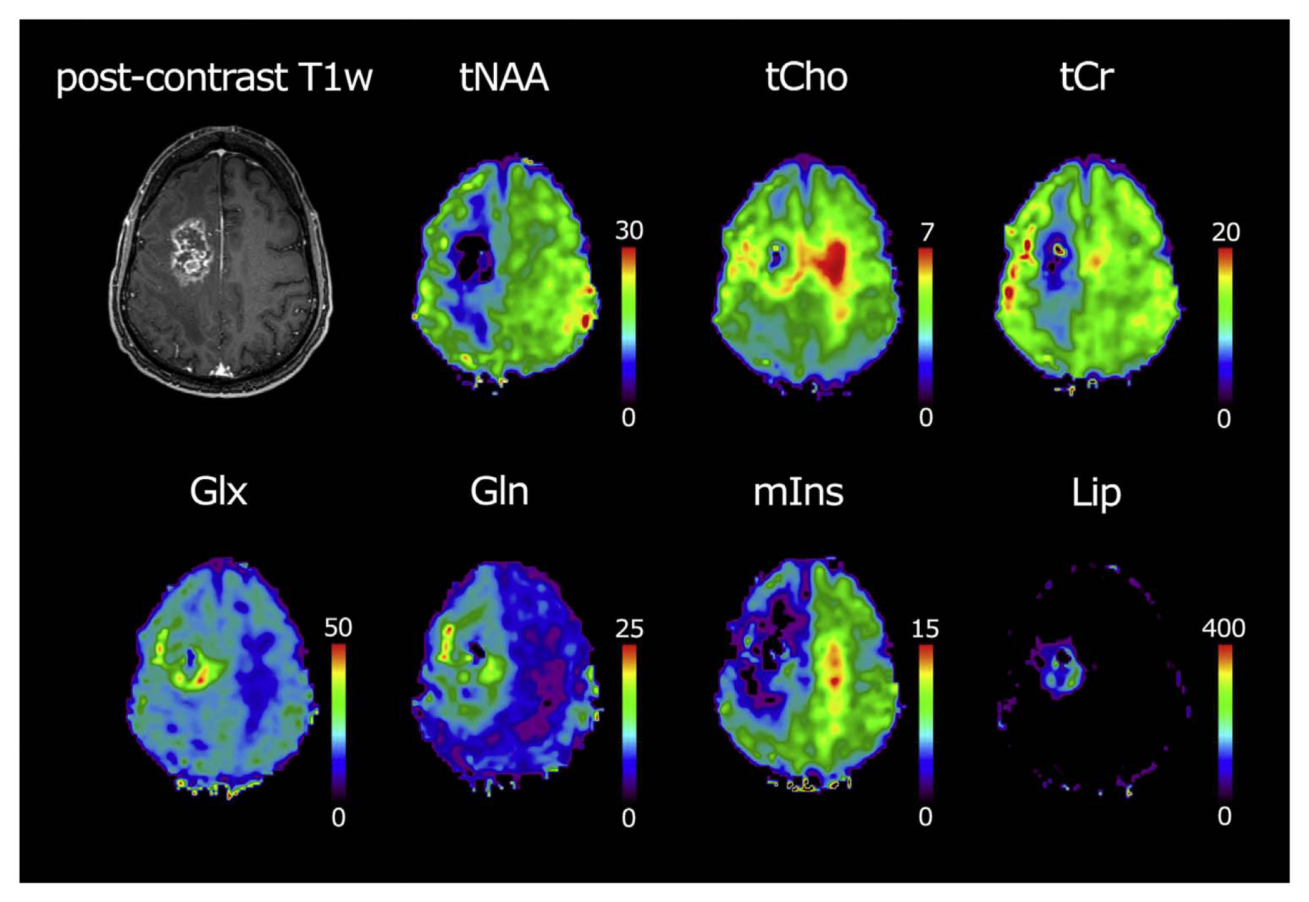
Data acquired via an ultra-short TE MRSI sequence at 7 T in a 38-year-old male patient with an anaplastic oligoastrocytoma WHO grade III. In the left top, a T_1_ weighted post-contrast image is shown. Sample maps of seven different brain metabolites are presented including total N-acetyl aspartate (tNAA), total choline (tCho), total creatine (tCr), glutamate (Glu), glutamine (Gln), myo-inositol (mIns), and sum of lipids (Lip). Note the increased tCho in the tumor border zone and contra-lateral side, decreased tNAA in the hemisphere containing the tumor, increased myo-inositol in the contralateral hemisphere, increased Glu and Gln in the tumor border zone, and increased lipids in the necrotic zone of the tumor.

Mapping of the neurochemical fingerprint of many brain pathologies, such as tumors, delivers spatially resolved information about metabolic activity, grade, or the distinction between recurrence and pseudoprogression during treatment (Fig. 5) (Heckova et al., 2016).

## Remaining challenges and future work

A number of challenges remain in clinical imaging at 7 T. The 3D - FLAIR and 3D - T2 sequences at 7 T are still problematic, as they are inherently more sensitive to B1 inhomogeneity and encounter SAR limitations, and because of the prolonged T1 relaxation times at 7 T in comparison to 3 T (Tallentyre et al., 2010), (Springer et al., 2016). Variable flip angle 3D sequences offer an alternative to conventional spin-echo T2-weighted sequences at 7 T, but in some cases need further optimization to reproduce the desired contrasts. The temporal lobes, for instance, have been found to be difficult to assess with a variable flip-angle FLAIR sequence at 7 T because of poor B1 efficiency (van Veluw et al., 2013; Visser et al., 2010). The 3D DIR sequence benefits from the use of higher resolution at 7 T, demonstrating (beneath the hyperintense signal of outer, glia-rich layers of the cortex) a slight decrease in the hyperintense signal of the cortex and lesions, of cortical lesions as well as WM lesions, compared to 3 T (Springer et al., 2016).

Optimization work still has to be conducted for the T1_FLASH sequence at 7 T to enhance gray/white matter contrast. The detection of intra- and extracellular methemoglobin, which is based on the assessment of a T1-weighted sequence, was nevertheless possible using the T1 FLASH sequence at 7 T (Springer et al., 2016).

The coil-related signal decrease in the posterior fossa poses a well known problem, which can be mitigated by the use of dielectric pads (O'Brien et al., 2013) and is likely to be overcome in a near future using parallel transmit technology.

In SWI, although there are existing methods for combining separate channel phase information, even in the absence of a reference volume coil (Parker et al., 2014; Robinson et al., 2011; Robinson et al., 2015) these have not been implemented by all vendors for online reconstruction. The implementation of automatic reconstruction on the scanner console could increase the applicability of SWI, or other methods using phase information, such as Quantitative Susceptibility Mapping (Deistung et al., 2016; Shmueli et al., 2009), in a clinical setting.

In summary, increased SNR through higher static magnetic field strength can be translated into higher spatial resolution or contrast, or can theoretically be invested into a reduction in scan time (particularly in spectroscopy). In some applications, increasing the resolution at 7 T (e.g. from 1.0 mm to 0.7 mm isotropical) may nonetheless lead to a slight increase in scan times, which may be considered worthwhile if it leads to an increase in diagnostic confidence. fMRI benefits from higher static magnetic field, which leads to more reliable presurgical brain tumor evaluation. The high potential of MRSI at UHF has already been shown in several clinical applications.
